# Evolving Skin Rash as a Rare Cutaneous Manifestation in a Pediatric Patient With COVID-19 Infection

**DOI:** 10.7759/cureus.26477

**Published:** 2022-06-30

**Authors:** Kedar Tilak, Karis Joung, Milena Apath

**Affiliations:** 1 Pediatrics, The Brooklyn Hospital Center, New York, USA; 2 Pediatrics, The Brooklyn Hospital Center, Brooklyn, USA

**Keywords:** infection, pediatrics, skin, rash, covid-19

## Abstract

COVID-19 has been one of the common infections that have affected numerous children across the world in the last two years. The clinical manifestations of this virus are varied, ranging from being asymptomatic to affecting multiple organ systems. There is a lot of ongoing research to find out the different manifestations that this infection can have in both adults and children. As with any viral illness, skin is one of the most commonly affected organs, and many viral illnesses can present with a rash. In our case, we found it interesting that our patient who tested positive for COVID-19 had a rash that began on the day of her initial presentation and evolved over time as the disease progressed, and hence, we thought it was important to highlight this rare case presentation as a cutaneous finding in children with COVID-19 infection.

## Introduction

The new SARS-CoV-2-induced disease (COVID), which began in Wuhan City, China, in December 2019, soon spread around the world and was declared to be a pandemic on March 11, 2020. Clinical signs and symptoms, disease course, and severity differ in pediatric patients as compared to adults. Viral infections are known to be associated with exanthems or rashes as they run their course through the body, and the coronavirus has also been known to cause dermatological manifestations in children [1]. In the following text, we report an interesting case of a 14-year-old female diagnosed with COVID-19 who presented with a rash over her arms on the initial day of her presentation to the hospital and evolved through the course of her illness, having a strikingly different appearance by day 3 of her illness. This evolution of the rash was thought to be an interesting skin finding in the pediatric population and hence prompted us to report it here.

## Case presentation

**Figure 1:**
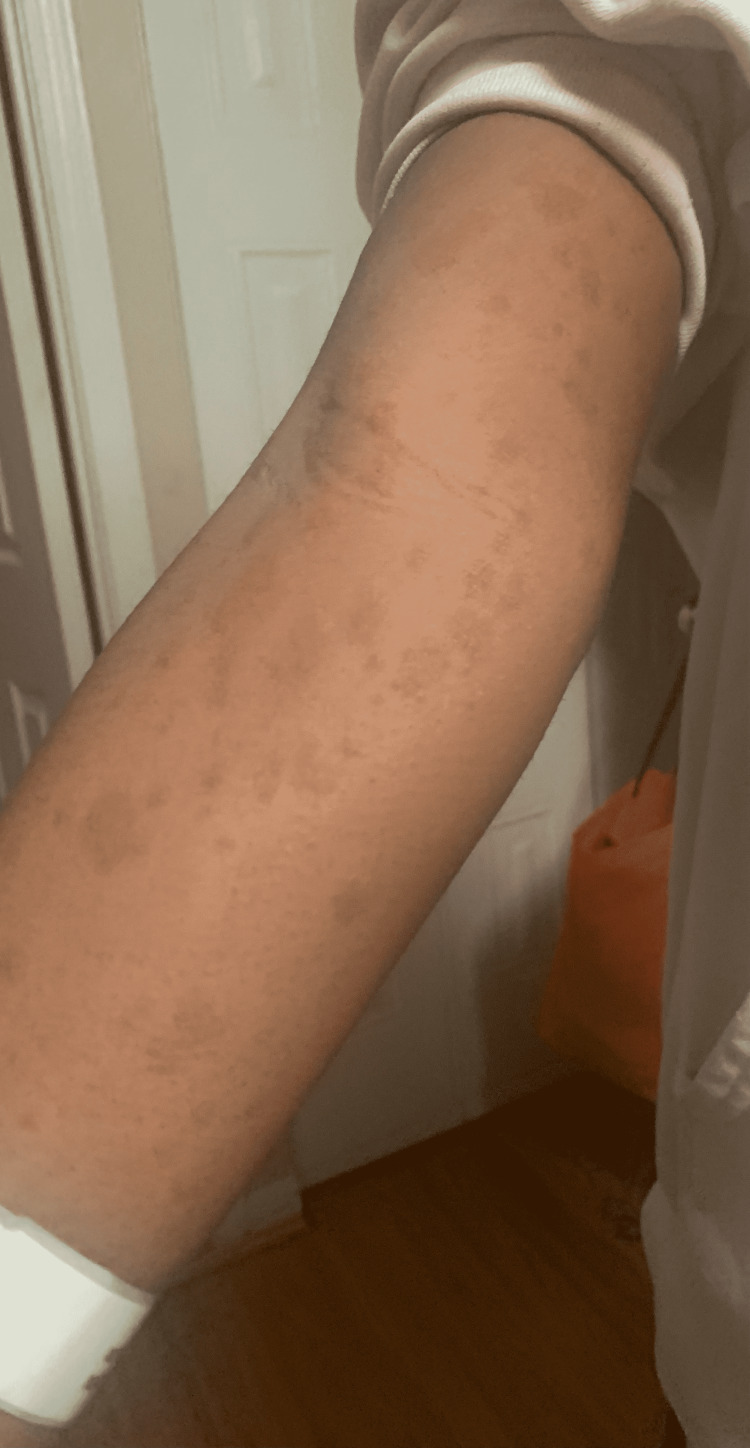
Initial rash

A 14-year-old female with a history of scoliosis and ADHD presented to our clinic for follow-up after being hospitalized for seven days for a COVID-19 infection. Initially, she had a worsening of her back pain and sore throat with some diarrhea, which prompted her to see her pediatrician. At the clinic, the patient was febrile (T 102F) and COVID-19 PCR was done. The evening after the clinic visit, she started to develop a rash with irregularly shaped, discreet erythematous macules and papules (Figure 1), which prompted her to visit urgent care and she eventually went to the ED.

While in the ED, the patient tested positive for COVID-19, was given dexamethasone and ibuprofen, and was discharged home. Later that same day, the rash became more pruritic and the mother called the ED and was instructed to give Benadryl at home. The medication helped with the itching, but the mother was concerned and later brought the patient to the ED. She was eventually admitted to the pediatric floor for further management as she developed skin findings and needed frequent monitoring of her respiratory status to prevent any compromise. On the day of admission, a respiratory viral panel was done and all were negative. Her initial labs have been summarized in Table 1. A blood culture was also sent, which showed no growth for five days. Elevated AST, ALT, and total and direct bilirubin are consistent with transaminitis and cholestasis secondary to acute COVID-19 infection. Labs were sent to rule out other viral causes: hepatitis B surface antigen, hepatitis C virus antibody, Ebstein Barr Virus (EBV) Ab, and hepatitis A IgM, which were all negative. Ultrasound of the abdomen showed a "coarsened hepatic echotexture," a non-specific indicator of hepatocellular diseases. There was no evidence of biliary obstruction or choledocholithiasis. Gallbladder wall thickening with trace pericholecystic fluid can be seen in cholecystitis. The patient was also started on vancomycin and ceftriaxone.

**Table 1:** Laboratory values for the patient at initial presentation

Test		Results
Complete blood count	Total WBC	15,100
	Neutrophils	74%
	Monocytes	2%
	Lymphocytes	9%
	Basophils	13%
	Hemoglobin	11 mg/dL
	Platelets	2,48,000
Comprehensive Metabolic Panel	Sodium	134
	Potassium	3.6
	Chloride	99
	CO_2_	21
	Creatinine	0.6
	BUN	6
	Calcium	8.2
	Total bilirubin/direct	4.5/2.74
	Alkaline Phosphatase	155
	AST/ALT	243/258
	GGT	100
Coagulation Panel	aPTT/PT/INR	28.7/13.3/1.1
Acute Phase reactants	ESR	>130
	CRP	124
	Ferritin	1287.7
	BNP	472
	Troponin I	2.09
	D-dimer	177
	Fibrinogen	746

**Figure 2:**
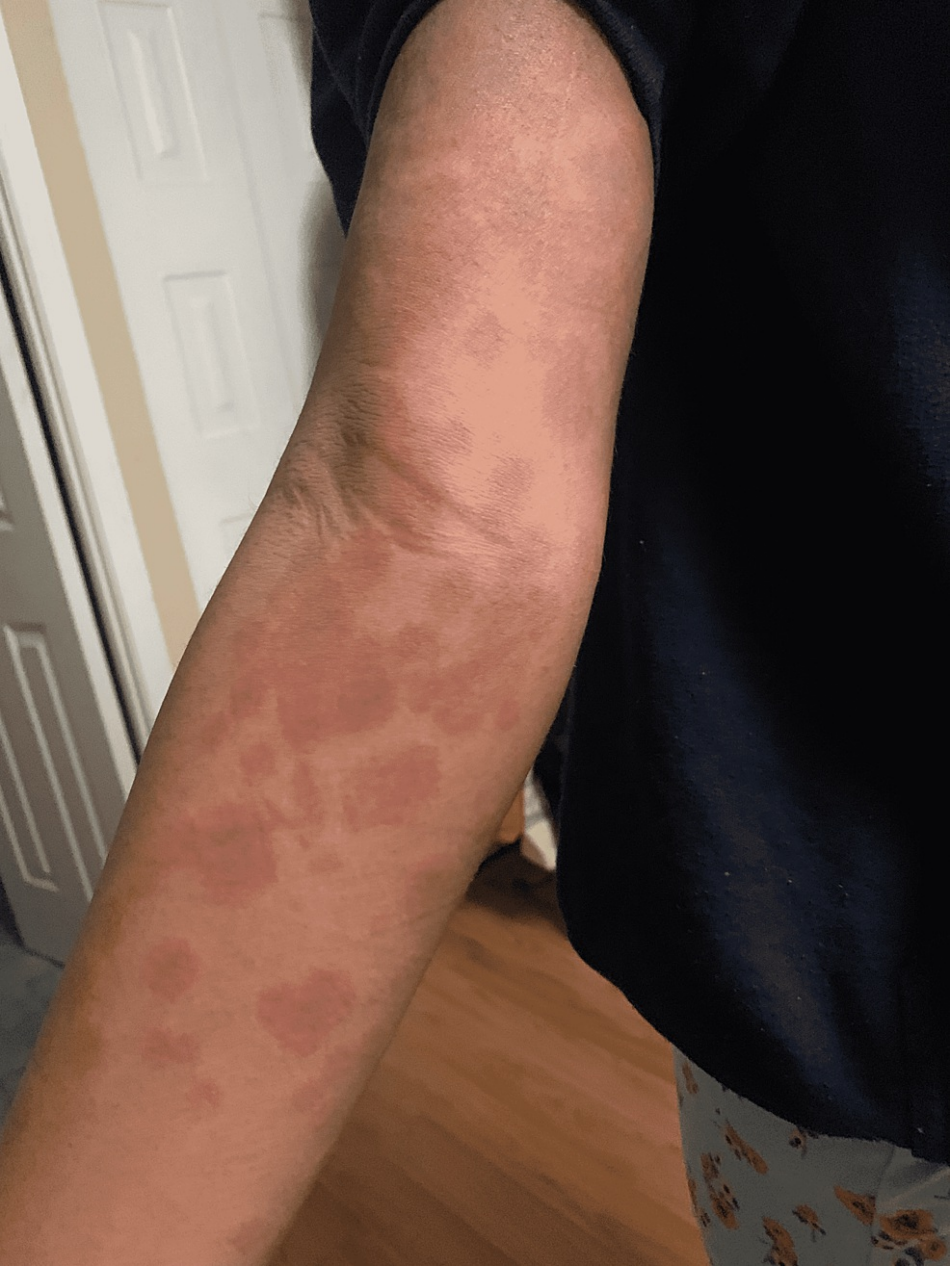
Rash as it progressed

On hospital day 2, the patient was initiated on Enoxaparin prophylaxis 40 mg subcutaneously as her D-dimer levels were up trending (4070→ 4524→ 4601). The decision was made to give Enoxaparin for one month. The patient also had persistently low blood pressure and was given a normal saline bolus and intravenous fluids. She still continued to have low blood pressure. The patient was transferred to the PICU to be started on an epinephrine drip. At that time, an electrocardiogram (EKG) showed diffused ST elevations. A cardiac echocardiogram was also done for her, which showed good ventricular function with no evidence of visible valvular vegetation or pericardial effusion. At this time, she had a change in the appearance of her rash, and her rash, which was initially in the form of macules and papules, now progressed and could be described as confluent erythematous to dark purple patches (Figure 2). She also had mild respiratory distress and a chest X-ray was done which showed "subtle peripheral and basilar parenchymal opacities, which may represent atelectasis" and she was put on a high-flow nasal canula for respiratory support but weaned off to room air as tolerated.

On hospital day 3, the patient received one dose of tocilizumab and started on Remdesivir, which was continued for five days, and dexamethasone to complete a 10-day course of steroids. Her antibiotics were switched to amoxicillin - 2 g every 12 hours to complete a total of 10 days of antibiotics. On hospital day 7, the patient was clinically stable for discharge and sent home with enoxaparin for one month, amoxicillin to complete a 10-day course, prednisolone to complete a 10-day course, and iron, magnesium, and calcium supplements. No further symptoms developed after the patient was discharged home and the rash resolved.

## Discussion

The COVID-19 viral pandemic that began in 2020 has spread across the world and has affected patients in a variety of different ways. The infection in children can range from being completely asymptomatic to having multisystem inflammatory syndrome (MIS-C), which can also result in death. The manifestations of the disease can affect multiple organ systems, and the skin is one of the most commonly affected organs.

Andina et al. [1-3] published three review articles during the pandemic, and their studies have described the various skin manifestations that COVID-19 can have in children and their associated histological findings. In their initial study that was published in November 2020 [1], they reported that chilblains were more frequently seen in children and young patients. Chilblains [4] have been described as inflammatory lesions that affect the acral regions and persist for more than one day. They can be described as erythematous and oedematous macules and nodules, which can sometimes present as ulcerated plaques on the dorsal surface of fingers and toes and are triggered by cold. These lesions, when associated with viral symptoms, have been collectively referred to as "COVID toes" [5]. On reviewing the histology of these lesions, they had moderate to severe perivascular lymphocytic infiltrate [3].

In the subsequent study published by Andina et al. in November 2020 [2], they described erythema multiforme (EM), urticaria, and Kawasaki disease-like inflammatory multisystemic syndrome with its associated skin manifestations. Erythema multiforme can be described as a symmetrical skin eruption with erythematous lesions, which are also referred to as iris or target lesions. EM lesions have been described in both adults and children with COVID-19 infection [6]. A case report published by Torrelo et al. [7] describes four children with chilblain-like lesions who also had associated EM, with both target and targetoid lesions; one of the four patients had a positive PCR test result for SARS-CoV-2; two of the cases had endothelial positive immunohistochemistry stain for SARS-CoV-2 spike protein on skin biopsy.

Urticaria is a skin condition that usually manifests as itchy, circumscribed, and raised wheals and was seen in 10-20% of the COVID-19 patients with cutaneous manifestations [6]. In a systematic review published by Imbalzano et al. [8], viral infections have been described as the most common cause of urticaria in children, with parvovirus, rhinovirus, EBV, hepatitis viruses, and HIV being the more common ones. The study also talks about a vesicular exanthem that can be seen in patients with COVID-19. This looks similar to the papulovesicular eruption that is seen in patients with varicella [9]. This study published by Marzano et al. described these lesions as appearing three days after the onset of systemic symptoms and disappearing by day 8 without scarring.

One of the cases described by Joob and Wiwanitkit [10] in Thailand describes a skin rash with petechiae that was thought to be secondary to dengue (high prevalence in the area). The patient further presented respiratory problems and then tested positive for COVID-19 infection via RT-PCR.

A few other rashes have also been described; for instance, Zhang et al. have described acro-ischemia [11], which is the cyanosis of the fingertips and toes with skin bullae and dry gangrene, and he also describes a possible association of hypercoagulable states in patients with COVID-19. Another article published by Bouazuz et al. [12] talks about patients with COVID-19 having a livedo-like rash with necrotic purpura.

The feature that makes our case unique is that the patient had a rash that started off as erythematous macules and papules and then progressed to dark purple patches with her disease. We searched for reported cases where pediatric patients diagnosed with COVID-19 had a rash that was changing its character through the course of the disease and were unable to find any reported cases with a similar course.

## Conclusions

COVID-19 is a new infection and it has been shown to have a variety of clinical manifestations. Currently, there are multiple studies around the world describing the clinical course of this infection. A variety of cutaneous manifestations have also been reported and, as per recent studies, these can aid in early recognition and better prognosis in COVID-19 patients. Further research is warranted for a detailed and full analysis of COVID-19-associated skin manifestations.
